# Causes, Complications and Short-Term Outcome of Acute Kidney Injury in a Resource-Limited Setting

**DOI:** 10.1155/ijne/4484755

**Published:** 2024-12-24

**Authors:** Nalaka Herath, Shamila De Silva, Prasitha Liyanage, Sameera Kumara, Suganthika Devi, Vajira Abeysekara, Ruvini Mallawarachi, Suharshi Perera, Iresha Karunathilaka, Sameera Samarasinghe, Kosala Weerakoon

**Affiliations:** ^1^Department of Nephrology, Colombo North Teaching Hospital, Ragama, Sri Lanka; ^2^Department of Medicine, Faculty of Medicine, University of Kelaniya, Ragama, Sri Lanka; ^3^Department of Parasitology, Faculty of Medicine and Allied Sciences, Rajarata University of Sri Lanka, Anuradhapura, Sri Lanka

**Keywords:** acute kidney injury, chronic kidney disease, haemodialysis, leptospirosis, Sri Lanka

## Abstract

**Aims:** The outcome of acute kidney injury (AKI) depends on causes, patient factors and care received. We studied the causes, complications and 90-day outcomes of patients with AKI at a tertiary referral centre in Sri Lanka.

**Methods:** Patients aged 18 years or older with AKI referred to nephrology services were analysed retrospectively. AKI severity was assessed using the KDIGO classification. Information was gathered from hospital and clinic records.

**Results:** Of the 464 patients studied, 262 (56.5%) were males. The mean age of the study sample was 57.04 (SD 16.85) years. The majority (212–45.69%) were discharged with normal renal functions, 173 (37.28%) were discharged with impaired functions, and 79 (17.03%) died during hospital stay. There were 377 patients at 3 months follow-up; 331 (87.8%) had normalised renal function, 40 (10.6%) had not recovered fully and 6 (1.6%) had succumbed. Progression of AKI to chronic kidney disease or death was significantly high in patients aged > 60 years (*p*=0.017). More severe AKI was associated with type 2 diabetes (*p*=0.0042), hypertension (*p* < 0.0001) and multiple comorbidities (*p*=0.0014). Persons with no comorbidities had less severe AKI (*p*=0.0004). Even in the early stages of AKI, there was significantly high mortality (11% in AKI stages 1 and 2) which doubled in stage 3 (22%). Mortality was low in patients with prerenal causes of AKI (OR: 0.59, 95% CI: 0.35–0.99 and *p*=0.047).

**Conclusions:** AKI in elderly and comorbid patients has high morbidity and mortality. Identification of individuals who are at high risk of developing AKI is important for its prevention, early diagnosis and proper treatment. Limitations in infrastructure, manpower, local research, reporting and recording of AKI are key challenges in providing optimal care for AKI in Sri Lanka.

**Summary**

• Acute kidney injury causes high morbidity and mortality. Early diagnosis is vital to prevent the progression and development of life-threatening complications.

## 1. Background

Acute kidney injury (AKI) is defined as an abrupt reduction in kidney function, which includes both injury (structural damage) and impairment (loss of function) [1]. As estimated by the International Society of Nephrology, there are 13.3 million cases of AKI occurring annually worldwide, leading to 1.7 million deaths. Almost 11.3 million cases of AKI take place in resource-limited settings, particularly in low- and middle-income countries [2].

AKI can develop in community-dwelling adults as well as in hospitalized persons. It is seen in approximately 20% of the patients admitted to hospital for other reasons. Development of AKI increases the likelihood of death almost four-fold [3]. Over 2.3 million people worldwide die from it annually (ISN 0 by 25 Global Snapshot Project) [2]. AKI is multifactorial in aetiology and has well-known complications and consequences [1]. It is also known to progress to chronic kidney disease (CKD) and end-stage kidney disease (ESKD) over time [4].

Though publications on the causes of hospital-acquired AKI are lacking, there is a variety of publications on community-acquired AKI in Sri Lanka [5]. Factors such as poor socioeconomic status and environmental and occupational exposures play a major role in the development of AKI. Tropical infections, diarrhoeal illnesses, and exposure to animal, plant, and environmental toxins are some causes of community-acquired AKI in Sri Lanka. Leptospirosis is hyperendemic with an estimated morbidity of 300.6 and mortality of 17.98 per 100,000 population per year [4]. Russell's viper (*Daboia russelii*) and hump-nosed pit viper (*Hypnale* spp.) account for many community-acquired AKI in Sri Lanka. Intentional or accidental exposure to toxins (such as yellow oleander), substances (such as paracetamol, paraquat and organophosphates) and oxalate nephropathy are recognized causes of community-acquired AKI in the country [5].

The outcome of AKI varies and depends on the cause of AKI, patient factors such as age and comorbidities and the care received in the hospital. Facilities for optimal management of AKI differ between higher- and lower-income countries; in the latter, access to emergency haemodialysis may not be easily available and this has a direct impact on outcome [6]. The patients who require renal replacement therapy (RRT) need prolonged hospitalization. Many cases of AKI are avoidable. The International Society of Nephrology launched an initiative in 2013, aiming to improve the timely diagnosis and treatment of AKI and to eliminate preventable deaths from AKI globally by 2025 [2].

Data on causes, associations and outcomes of AKI, from South Asia as a whole and Sri Lanka in particular, are lacking. There is inadequate research into all aspects of the epidemiology of AKI in Sri Lanka [5]. We studied the causes, complications and 90-day outcomes of patients with AKI at a tertiary referral centre in Sri Lanka.

## 2. Methods

This retrospective study was carried out at the Colombo North Teaching Hospital (CNTH), Ragama. The town of Ragama is situated 16 km north of Colombo, the commercial capital of Sri Lanka. Ragama is a bustling multiethnic, multicultural town with a teaching hospital, a medical faculty, and a large central railway station. The CNTH is the largest hospital in the Gampaha district and receives patients from most of the district as well as from adjoining districts.

The study was conducted at the Nephrology Unit of the CNTH, which receives referrals from various units of the 1750-bed tertiary care hospital. Patients aged 18 years or older with AKI referred to the Nephrology Service of the CNTH over 15 months from May 2018 were retrospectively studied using hospital records. The severity of AKI was classified using the kidney disease improving global outcomes (KDIGO) AKI classification [7]. A data-gathering instrument was used to gather information from hospital and clinic records. Patients were anonymised for data collection. The outcome was recorded as complete or partial renal recovery or death at discharge and 90 days post discharge. Complete renal recovery was defined as the recovery of renal function to eGFR > 60 mL/min/1.73 m^2^, while partial renal recovery was defined as eGFR < 60 mL/min/1.73 m^2^.

Baseline serum creatinine was defined as the most recent outpatient serum creatinine done 7–365 days before the current admission. If this value was not available, the most recent inpatient serum creatinine value from the index AKI admission was used. ICD coding was not used in the identification of AKI. Biopsies were not performed in any of the patients.

Data were entered in MS Excel (Microsoft Corporation and Microsoft Excel) and logical and random checks were done. Statistical analysis was carried out using GraphPad Prism Version 9.3.1 (GraphPad Software, LLC) and MS Excel. A *p* value of < 0.05 was considered statistically significant. Categorical data were analysed and presented in terms of frequency and percentages, with 95% confidence intervals (95% CIs). Continuous data were described using mean, standard deviations (SDs) and 95% CI. Group comparisons were made using Mann–Whitney *U* test and Pearson's Chi-square test, for continuous and categorical variables, respectively.

This study was approved by the Ethics Review Committee of the Faculty of Medicine, University of Kelaniya, Sri Lanka (P 222/12/2018).

## 3. Results

A total of 464 patients were studied; 262 (56.47%) were male. The mean age of the study sample was 57.04 (SD 16.85) years (Supporting Information 1: baseline characteristics of the study group).

Most patients were referred from general medical units (*n* = 291, 62.72%), followed by general surgical (*n* = 134, 28.88%) and urology (*n* = 11, 2.37%) units.

### 3.1. Clinical Outcomes

A total of 212 patients (45.69%) were discharged with normal renal function while 173 (37.28%) were discharged with impaired renal function. There were 79 deaths (17.03%) during the initial hospital stay (Table 1). At 3 months, in 331 patients, renal function had become normal while 40 had not recovered from AKI completely and 85 had succumbed to illness.

Most deaths (93%) were during acute illness (Table 1). Even in the early stages of AKI, there was significantly high mortality (11% in AKI Stages 1 and 2) which doubled in Stage 3 to 22%.

### 3.2. AKI Stages, Clinical Outcomes and Age

The severity of AKI was classified as KDIGO AKI Stage 1 in 65 (14.19%), Stage 2 in 85 (18.56%) and Stage 3 in 308 (67.25%) patients. At three months, 127 (27.41%) patients had either developed CKD or died (Table 1). AKI progression, development of CKD and death was significantly high (*p*=0.017) in patients older than 60 years. (Table 2).

### 3.3. AKI Causative Factors

The common causative factors for AKI were sepsis (*n* = 232, 49.89%), medications (*n* = 149, 32.04%), dehydration (*n* = 110, 23.65%), leptospirosis (*n* = 31, 6.66%) and Russell's viper and humped nose viper bites (*n* = 26, 5.59%) (Table 3). The mortality was low in patients with prerenal causes of AKI (OR: 0.59, 95% CI: 0.35–0.99 and *p*=0.047).

### 3.4. Comorbidities and AKI Outcomes

The analysis of comorbidities associated with the severity of AKI is shown in Table 4. Most of the study group had one or more comorbid diseases. Type 2 diabetes was the commonest comorbidity, present in 176 patients (37.93%), followed by hypertension in 165 (35.33%), ischaemic heart disease in 59 (12.73%) and chronic liver cell disease in 28 (6.0%). Multiple comorbidities were present in 157 (33.62%) patients while 210 (44.97%) patients did not have any comorbidities (Table 4).

More severe AKI (stage 3) was associated with diabetes (OR: 1.8447, 95% CI: 1.2124–2.8068 and *p*=0.0042), hypertension (OR: 6.2351, 95% CI: 4.0753–9.5397 and *p* < 0.0001) and multiple comorbidities (OR: 2.058, 95% CI: 1.3226–3.2035 and *p*=0.0014). Patients with no comorbidities had less severe stages of AKI (OR: 0.4797, 95% CI: 0.3201–0.7191 and *p*=0.0004).

### 3.5. Indications for Haemodialysis

As this study did not include ICU patients, intermittent haemodialysis was the only mode of RRT. Uraemia, hyperkalaemia, severe acidosis and fluid overload were considered definitive emergency indications for haemodialysis. Some patients underwent haemodialysis for severe AKI before they developed life-threatening complications of AKI needing emergency haemodialysis.

Overall, 122 (26.3%) patients underwent haemodialysis as a RRT for uraemia, hyperkalaemia, severe acidosis, fluid overload and severe AKI (Table 5).

## 4. Discussion

This retrospective study evaluated 464 patients developing AKI over 15 months in a tertiary referral centre in a lower middle-income country. Most patients were elderly males with one or more comorbidity. Type 2 diabetes was the most common comorbidity followed by hypertension. Mortality was higher in older patients, those with severe AKI and patients having one or more comorbidities. The most common aetiological factors were sepsis, medications and dehydration. Increased severity of AKI and primary outcome of CKD or death were commoner in older patients and in those with comorbidities.

AKI remains a common clinical problem in the elderly due to reduced renal reserve, higher frequency of nephrotoxin usage and polypharmacy [8]. In this study, the mean age of the sample was 57 years, which is relatively young compared with findings from across the globe. Liaño and Pascual reported a mean age of 64 years in a study of AKI episodes occurring in adult patients admitted to 13 tertiary care hospitals in Madrid [9]. The mean age of patients was 73 years in two UK district hospitals, as reported by Meran et al. [10], and 64.7 years in a Canada-based ICU study [11]. Similarly, a study done in Singapore showed a median age of 65.8 years [12]. The relatively younger age of our study participants may be due to community-acquired causes of AKI such as leptospirosis, snakebite and sepsis. These occupational causes are more commonly seen in younger people. The rising prevalence of type 2 diabetes in Sri Lanka, including among younger people, may have contributed to the sample's lower mean age, as diabetes is an independent risk factor [13].

While there were multiple causes of AKI, sepsis is the most common causative factor, which was seen in nearly half the study population. Other common causes were medication and dehydration. The causes overlapped in some patients. In many patients, late presentation, delay in commencing appropriate treatment for sepsis and pre-existing kidney diseases increase the risk of developing AKI [14]. Liaño and Pascual describe the most frequent causes of AKI in Spain as acute tubular necrosis (ATN), prerenal AKI, acute-onset chronic renal failure and obstructive AKI [9]. Sepsis was the most common cause of AKI, seen in 40%–50% of critically ill patients in a study done by Gómez and Kellum in the USA [15]. Wang et al. found that in China, 45%–75% of AKI episodes were associated with sepsis [16]. In a study done by Shum and colleagues, 49.2% had sepsis-induced AKI [17].

Nephrotoxic drugs were the cause of a third of the AKI episodes in our study. Nonsteroidal anti-inflammatory drugs (NSAIDs), continuation of angiotensin-converting enzyme inhibitors (ACEIs) and angiotensin receptor blockers (ARBs) in dehydrated hypotensive patients were the commonest culprits. A multicentre cross-sectional study from China found that antibiotics, diuretics and proton pump inhibitors play a major role in drug-induced AKI [18]. Ghane Shahrbaf and Assadi found that aminoglycoside antibiotics, NSAIDs, contrast agents and ACEIs were the most common causes of AKI in hospitalized patients [19]. Avoiding nephrotoxic medication in high-risk patients or early withdrawal of such drugs is the best method of reducing drug-induced AKI. Educating junior members of healthcare teams, who the patients encounter first, is important in this regard.

We identified prerenal causes as a precipitant of AKI in 23.65% of the cohort. A study by Tang et al. reported that prerenal causes accounted for 49.1% of AKI in medical departments [20]. Teo et al. reported a prerenal cause of AKI in 27.7% of the participants in their study [12]. A study done by Goyal et al. showed that 21% of AKI were due to prerenal causes [21]. Volume resuscitation and restoration of the baseline volume status are crucial in the event of true extracellular volume depletion. Knowledge of baseline weight, serial weight measurement, careful attention to intake and output and frequent volume status assessment direct the strategy for resuscitation [14].

Malaria was not identified as a cause of AKI in this cohort since malaria has been eradicated in Sri Lanka since 2012 [22]. Obstetric causes of AKI were also not seen in this cohort, as they are most likely to be managed at ICU and this study did not include ICU patients.

The incidence and prevalence of noncommunicable diseases are rising, and the outcome of AKI is different in those with various comorbid conditions. Patients with no comorbidities had the best outcome, while those with type 2 diabetes had high rates of CKD or death compared with other comorbid conditions. Multiple studies have shown that diabetes alone is an independent risk factor for AKI [23]. Farooqi and Dickhout showed that the risk of AKI increased in patients with diabetes [24], while a study done in Singapore by Teo et al. showed that diabetes and hypertension were the commonest comorbidities [12]. Therefore, special attention to patients with diabetes is important as AKI in such patients is likely to have an adverse outcome.

This study highlights the following shortcomings directly related to standard renal care. Sri Lanka has 1.6 nephrologists per million population [5], which is highly inadequate to maintain continuous renal services. This compares with 7.4 and 20.1 nephrologists per million population in the United Kingdom and Europe, respectively [5]. More trainees should be incorporated into nephrology training in Sri Lanka to address the scarcity of professionals in the field. The lack of knowledge of AKI among healthcare staff often leads to late diagnosis and late referral to nephrology services. Sometimes patients seek treatment from traditional healers residing within the local community, significantly delaying standard healthcare.

Expansion of public awareness programs on the prevention of common community-acquired AKI, including avoidance of common nephrotoxic drugs, toxin exposures and prevention of dehydration, is an immediate need. Early presentation to medical services in case of leptospirosis and snake bite can significantly reduce morbidity and mortality due to AKI. Drug level monitoring, which is not routinely available in Sri Lanka, is essential when administering potentially nephrotoxic antibiotics such as vancomycin, gentamicin and amikacin. The establishment of an AKI registry would be helpful to know the incidence, causes and complications of AKI and for the fair allocation resources to the different areas of the country. More research should be done for the prevention and early diagnosis of common causes of AKI in Sri Lanka.

### 4.1. Limitations

As this was a retrospective study, lack of completeness of medical records was a limitation.

Eight patients were lost to follow-up at 3 months. The unavailability of the most recent outpatient serum creatinine 7–365 days before the current admission in some patients is another limitation. The generalizability of data is also a potential limitation.

## 5. Conclusions

AKI in elderly and comorbid patients has high morbidity and mortality in Sri Lanka. Identification of individuals who are at high risk of developing AKI is important for its prevention, early diagnosis and proper treatment. The limitations in the infrastructure, manpower, local research, reporting and recording of AKI are key challenges in providing optimal care for AKI in Sri Lanka.

## Figures and Tables

**Table 1:** Clinical outcomes on discharge and at 3-months follow-up.

Outcomes on discharge	*N* = 464	%
Recovered	212	45.69
Residual renal impairment	173	37.28
Death	79	17.03

**Outcomes at 3 months**	**N**=377^†^	**%**

Recovered	331	87.80
CKD	40	10.60
Death	6	1.60

Abbreviation: CKD, chronic kidney disease.

^†^Lost to follow = 8 and previous deaths = 79.

**Table 2:** Association between AKI stage, clinical outcomes and age.

AKI stage and clinical outcomes		Age categories (%)		Crude OR	95% CI	*p*
		≥ 60 Years	< 60 Years			
AKI stage	3	169 (55)	139 (45)	1.90	1.28–2.82	0.0016
	1 and 2	59 (39)	92 (61)			

Outcome at 3 months	Not recovered^†^	72 (58)	52 (43)	1.66	1.09–2.53	0.017
	Recovered	149 (45)	179 (55)			

Abbreviations: AKI, acute kidney injury; CI, confidence interval; OR, odds ratio.

^†^Not recovered = death or chronic kidney disease (CKD).

**Table 3:** Association between AKI stage and potential causative factors.

Causative factor		Outcomes (%)		Crude OR	95% CI	*p*
		AKI-1/2	AKI-3			
Pre-renal	Negative	114 (33)	229 (67)	1.0629	0.6774–1.6678	0.7907
	Positive	37 (32)	79 (68)			

Medication	Negative	104 (32)	220 (68)	0.8851	0.5792–1.3525	0.5726
	Positive	47 (35)	88 (65)			

Sepsis	Negative	78 (35)	146 (65)	1.1856	0.8029–1.7506	0.3919
	Positive	73 (31)	162 (69)			

Pyelonephritis⁣^∗^	Negative	136 (33)	270 (67)	1.2425	0.6589–2.3431	0.5024
	Positive	15 (29)	37 (71)			

Leptospirosis	Negative	143 (33)	286 (67)	1.375	0.5973–3.1651	0.4541
	Positive	8 (27)	22 (73)			

Snake bite	Negative	144 (33)	289 (67)	1.3524	0.5557–3.2913	0.5058
	Positive	7 (27)	19 (73)			

Other	Negative	141 (33)	283 (67)	1.245695	0.5821–2.6652	0.5715
	Positive	10 (29)	25 (71)			

Abbreviations: AKI, acute kidney injury; CI, confidence interval; OR, odds ratio.

⁣^∗^clinical and ultrasound diagnosis.

**Table 4:** Association between AKI stage and comorbidities.

Co-morbidities		Outcomes (%)		Crude OR	95% CI	*p*
		AKI-1/2	AKI-3			
Type 2 diabetes	Negative	108 (38)	177 (62)	1.8447	1.2124–2.8068	0.0042
	Positive	43 (25)	130 (75)			

Hypertension	Negative	113 (38)	183 (62)	6.2351	4.0753–9.5397	< 0.0001
	Positive	38 (23)	124 (77)			

Ischaemic heart disease	Negative	138 (35)	260 (65)	2.0788	1.0673–4.0492	0.0314
	Positive	12 (20)	47 (80)			

Peripheral vascular disease	Negative	150 (34)	293 (66)	7.1672	0.9335–55.0275	0.0582
	Positive	1 (7)	14 (93)			

Asthma	Negative	147 (34)	289 (66)	2.1619	0.7145–6.5410	0.1723
	Positive	4 (19)	17 (81)			

Stroke	Negative	150 (34)	297 (66)	5.0505	0.6405–39.8255	0.1243
	Positive	1 (9)	10 (91)			

Malignancy	Negative	146 (33)	300 (67)	0.6813	0.2126 to 2.1834	0.5184
	Positive	5 (42)	7 (58)			

Chronic liver cell disease	Negative	145 (34)	287 (66)	1.6841	0.6619–4.2851	0.274
	Positive	6 (23)	20 (77)			

None	Negative	82 (27)	218 (73)	0.4797	0.3201–0.7191	0.0004
	Positive	69 (44)	88 (56)			

Multiple	Negative	116 (38)	190 (62)	2.0583	1.3226–3.2035	0.0014
	Positive	35 (23)	118 (77)			

Abbreviations: AKI, acute kidney injury; CI, confidence interval; OR, odds ratio.

**Table 5:** Association between AKI stages and indications for haemodialysis.

Indication for HD		AKI-1/2	AKI-3	Crude OR	95% CI	*p*
Uraemia	Negative	151	297	8.6572	0.4963–151.0037	0.1389
	Positive	0	8			

Acidosis	Negative	147	269	5.0548	1.7671–14.4597	0.0025
	Positive	4	37			

Hyperkalaemia	Negative	144	266	3.0934	1.3513–7.0817	0.0075
	Positive	7	40			

Anuria/Oliguria	Negative	147	249	11.2169	3.4510–36.4589	0.0001
	Positive	3	57			

Fluid overload	Negative	151	293	13.937	0.8229–236.0561	0.068
	Positive	0	13			

Severe AKI	Negative	145	254	5.1378	2.1573–12.2361	0.0002
	Positive	6	54			

HD done	Negative	141	188	10.6875	5.0515–22.6115	< 0.0001
	Positive	8	114			

Abbreviation: HD, haemodialysis.

## Data Availability

Data and materials used in this work are available from the corresponding author upon reasonable request.

## Nomenclature

AKI: Acute kidney injury
CKD: Chronic kidney disease
CNTH: Colombo North Teaching Hospital
KDIGO: Kidney disease improving global outcomes
RRT: Renal replacement therapy

## Supporting Information

Supporting Information 1: Baseline characteristics of the study group.
