# Supplementary material for: Causes, Complications and Short-Term Outcome of Acute Kidney Injury in a Resource-Limited Setting
Source: Int J Nephrol. 2024 Dec 24;2024:4484755. doi: 10.1155/ijne/4484755 (PMC11688141; doi:10.1155/ijne/4484755)
Supplement: Supporting Information — Additional supporting information can be found online in the Supporting Information section. [file 4484755.f1.docx]

***Supplementary Material 1***

**Table: Baseline characteristics of the study group**

| **Parameter** | | **Number (%)** *N = 464* |
| --- | --- | --- |
|  |  |  |
| Gender | Male | 262 (56.47) |
|  | Female | 202 (43.53) |
| Age (Years) | Mean | 57.04 |
|  | SD | 16.85 |
| sCr on admission (µmol/l) | Mean | 327.11 |
|  | SD | 224.95 |
| Co-morbidities | Diabetes mellitus | 176 (37.93) |
|  | Hypertension | 165 (35.33) |
|  | Ischaemic heart disease | 59 (12.63) |
|  | Chronic liver cell disease | 28 (6.03) |
|  | Bronchial asthma | 21 (4.52) |
|  | Peripheral vascular disease | 16 (3.43) |
|  | History of stroke | 11 (2.36) |

*sCr – serum Creatinine, SD – Standard Deviation*
